# COVID-19 Compulsory Vaccination: Legal and Bioethical Controversies

**DOI:** 10.3389/fmed.2022.821522

**Published:** 2022-02-02

**Authors:** Filippo Gibelli, Giovanna Ricci, Ascanio Sirignano, Domenico De Leo

**Affiliations:** ^1^Section of Legal Medicine, School of Law, University of Camerino, Camerino, Italy; ^2^Department of Diagnostics and Public Health, Section of Forensic Medicine, University of Verona, Verona, Italy

**Keywords:** vaccinations, consent, obligations, autonomy, public health

## Abstract

The imposition of compulsory health treatments has always been a subject of animated legal and bioethical debate. What is at stake are two opposing interests that are in their own way protected by international treaties and constitutional provisions: the right to individual self-determination and the duty to defend and preserve collective safety. The global health crisis related to the COVID-19 pandemic has placed the issue of the legitimacy of imposing compulsory vaccination at the center of the multifaceted debate on pandemic health policies. Indonesia, Tajikistan, Turkmenistan, and the Federated States of Micronesia are currently the only four countries in the world where the COVID-19 vaccine is mandatory for all citizens. Italy was the first country in the European Union to introduce this obligation, effective from 8 January 2022 by virtue of the decree-law approved on 5 January 2022, which imposed vaccination compulsory for everyone over the age of 50. Similar paths have been undertaken by Greece and Austria, where the obligation will start respectively on 16 January 2022 (for citizens aged over 60) and 1 February 2022 (for citizens of all ages). However, in many civilized countries, “selective” forms of compulsory vaccination, i.e., aimed at specific categories of individuals, especially healthcare professionals, are already provided for. The present work aims to offer a concise and as much as possible exhaustive overview of the main ethical and legal issues related to compulsory COVID-19 vaccination, with reference to both the Italian and the international context, mainly European.

## Introduction

On 4 November 2021, the World Health Organization officially declared the entry into the fourth pandemic wave, identifying Europe as the epicenter of the new epidemic phase.

Although the proportion of the population fully vaccinated against COVID-19 is encouraging in industrialized countries (70% of the population in the US and Canada, 67% in South America, 64% in Asia and 62% in Europe have received at least one dose) (1), the impact of COVID-19 vaccination hesitancy could be a major hindrance to this delicate phase of the pandemic fight.

The international epidemiological trend has brought the issue of compulsory vaccination, temporarily neglected during the summer break, back to the attention of national institutions.

COVID-19 vaccination is already compulsory in many countries for specific categories of workers, mostly healthcare professionals, but a mandatory vaccination extended indiscriminately to the entire population is still largely unprecedented.

There are currently four countries in the world where COVID-19 vaccine is mandatory for all citizens: Indonesia, Tajikistan, Turkmenistan, and the Federated States of Micronesia.

On 5 January 2022, the Italian government approved a decree-law imposing compulsory vaccination for all citizens over the age of 50, which came into force on 8 January 2022. Italy was thus the first European country to adopt a form of compulsory vaccination extended to the entire population (albeit with a fixed age limit).

On November 19, 2021, Austrian Chancellor Alexander Schallenberg announced that in Austria the COVID-19 vaccine will be mandatory for all citizens from February 2022.

On 30 November 2021, Greek Prime Minister Kyriakos Mitsotakis declared that vaccination against COVID-19 will be compulsory in Greece from 16 January 2022 for all citizens over the age of 60.

Although the subject of hundreds of years of jurisprudential and bioethical reflection, the issue of the imposition of compulsory health treatment is still, in 2021, very far from seeing an unambiguous and shared key to interpretation.

Striking a fair balance between the protection of individual autonomy and the protection of collective health is in fact extraordinarily complex, especially when set in the peculiar epidemiological and scientific context of the COVID-19 pandemic.

This is because the COVID-19 vaccine has completely new features, from the technology used to make it to the particular way in which it combats the disease. This unique profile makes the discussion on compulsory vaccination particularly intriguing and raises legal and bioethical issues that have never before been addressed.

## At the Dawn of Compulsory Vaccination: the Fight Against Smallpox

The first compulsory vaccination policy in history dates back to the late 18^th^ century, during the American Revolutionary War, when General George Washington required his troops to be inoculated with the smallpox virus in 1777 (2).

Of all the diseases affecting the continental army, smallpox was one of the most fearsome threats, as it had a mortality rate of 10 to 60% in non-immune hosts.

According to historians' estimates, at the end of the 7-year war, nine times as many soldiers died of the disease consequences (63,000) as died in battle (7,000) (3).

Washington had the merit of recognizing the seriousness of the disease early on and devising an effective immunization strategy for his army, which gave his troops a significant physical and psychological advantage over their opponents.

A few years later, in 1796, English physician and naturalist Edward Jenner officially tested the first vaccine against smallpox by injecting a child's arm with a small amount of pus taken from the bumps of a woman suffering from cowpox, a form of smallpox that affects cows and, to a lesser extent, humans.

Jenner concluded that cowpox inoculation was a safe alternative to human smallpox virus inoculation and equally effective in terms of protection against smallpox disease (4).

After the scientific community recognized the efficacy and safety of the vaccine, the practice of smallpox vaccination spread widely in Europe, and several countries introduced mandatory vaccination requirements for their citizens, such as Norway in 1811, Russia in 1812 and Sweden in 1816 (5).

The first western nation to introduce free, universal, and compulsory smallpox vaccination was England with the Vaccination Acts of 1840, 1853 and 1867 (6).

The 1840 text provided for the smallpox vaccine to be free of charge and prohibited the variolation procedure, i.e., the inoculation of the subject to be immunized with human smallpox virus taken from an infected subject (the immunization technique practiced before Jenner's smallpox vaccine was developed).

The 1856 Act made vaccination compulsory for all children up to 3 months and established penalties for non-compliance.

The 1867 text tightened up the penalties for those who refused to vaccinate their children and introduced imprisonment for practicing variolation (7).

In the following decades, the outbreak of new smallpox epidemics triggered by the Franco-Prussian war prompted many European states to introduce compulsory vaccination.

In the United States of America, in 1905 the Supreme Federal Court issued a landmark judgement that legitimized the authority of states to “reasonably” violate personal liberties during a public health crisis by fining those who refused vaccination (8).

In the late 1960s, the World Health Assembly (WHA) initiated a strategic plan for the definitive eradication of the smallpox virus, which led to Resolution 11.54, adopted in 1958 by the Eleventh World Health Assembly (9).

On 1 January 1967, the World Health Organization launched the smallpox eradication programme, which led to the eradication of the virus in 1980.

The worldwide effort to combat the disease made it possible to eradicate a virus that was responsible for 500 million deaths between the XIX and XX centuries (10), mainly through compulsory vaccination.

## Compulsory Vaccination in the European Regulatory Context

The primary legal reference for the protection of fundamental human rights in the European regulatory context is the European Convention for the Protection of Human Rights and Fundamental Freedoms (ECHR), signed in Rome in 1950, in force since 1953, and adopted by the 47 member states of the Council of Europe (11).

Article 8 (“Right to respect for private and family life”) states that “*1. Everyone has the right to respect for his private and family life, his home and his correspondence. 2. There shall be no interference by a public authority with the exercise of this right except such as is in accordance with the law and is necessary in a democratic society in the interests of national security, public safety or the economic wellbeing of the country, for the prevention of disorder or crime, for the protection of health or morals, or for the protection of the rights and freedoms of others”*.

According to the Convention, therefore, forms of interference with the right to individual privacy are permitted whenever necessary to protect the public health of a democratic society.

This principle found a recent practical application in judgement no. 116/2021 of 8 April 2021 (Vavrička and others v. Czech Republic) by the European Court of Human Rights (ECtHR), which rejected the appeal of the parents of some Czech minors against national legislation prohibiting non-vaccinated children from enrolling in nursery school (12).

The Strasbourg Court interpreted the imposition of compulsory vaccination against the 10 vaccine-preventable childhood infectious diseases (diphtheria, tetanus, whooping cough, Haemophilus influenzae type b infections, poliomyelitis, hepatitis B, measles, mumps, rubella and–for children with specified health indications–pneumococcal infections) for admission to nursery school as a means of protecting public health, and as such not violating Article 8 of the ECHR.

The judgement sets out in detail the seven requirements that justify the interference in private life by national legislation:

1. The primary objective of compulsory vaccination must be to protect public health.
2. The imposition of compulsory vaccination must be based on a “*pressing social need”*, e.g., due to a low rate of spontaneous vaccination against a specific disease that could threaten public health.
3. “*Relevant and sufficient reasons”* are needed to impose mandatory vaccination.
4. The safety level of vaccines must be carefully evaluated in relation to scientific evidence.
5. The obligation cannot apply to persons with contraindications to the administration of the vaccine.
6. The obligation must be enforced through penalties for non-compliance, and may not provide for the forced administration of the vaccine.
7. The possibility for persons contesting penalties arising from non-compliance with the obligation to initiate appeals should be guaranteed.

Another important normative reference is represented by the Charter of Fundamental Rights of the European Union, signed in Nice in 2000 and legally binding for the European institutions and member states with the entry into force of the Treaty of Lisbon in 2007.

Article 1 (Human dignity) states: “*Human dignity is inviolable. It must be respected and protected”*.

Article 3 (Right to the integrity of the person) establishes: “*Everyone has the right to respect for his or her physical and mental integrity. In the fields of medicine and biology, the following must be respected in particular: the free and informed consent of the person concerned, according to the procedures laid down by law; the prohibition of eugenic practices, in particular those aiming at the selection of persons; the prohibition on making the human body and its parts as such a source of financial gain; the prohibition of the reproductive cloning of human beings”* (13).

The concept of free and informed consent expressed in Article 3 is borrowed from the Convention on Human Rights and Biomedicine (Oviedo Convention), the first international treaty on bioethics, signed in Oviedo (Spain) on April 4, 1997, and entered into force on December 1, 1999, following ratification by the first five member states of the European Union.

Article 5 of the Oviedo Convention states: “*An intervention in the health field may only be carried out after the person concerned has given free and informed consent to it. This person shall beforehand be given appropriate information as to the purpose and nature of the intervention as well as on its consequences and risks”* (14).

## The Legal bases of Compulsory Vaccination in the Italian Legal System

In the Italian legal system, the right to health is enshrined in the Constitution, which in Article 32 states that “*The Republic safeguards health as a fundamental right of the individual and as a collective interest, and guarantees free medical care to the indigent. No one may be obliged to undergo any health treatment except under the provisions of the law. Under any circumstances, the law may not violate the limits imposed by respect for the human person”* (15).

Therefore, health is not only a “*fundamental right of the individual”* but also a “*collective interest”*.

The Italian Constitution aims on the one hand to protect the individual's right to self-determination, and on the other to guarantee the health of the community.

The protection of public health may entail the imposition of compulsory health treatments, which would not be permitted under normal conditions, but which becomes legitimate if provided for by specific laws.

The Italian Constitutional Court, the main constitutional guarantee body, which is called upon to verify the conformity of state and regional laws and acts having the force of law with the Constitution, fully illustrated the concept of balancing the protection of the right to individual self-determination and the safeguarding of public health in judgement no. 307 of 22 June 1990, concerning the constitutional legitimacy of compulsory polio vaccination for children within the first year of life: “*… the law imposing a medical treatment is not incompatible with article 32 of the Constitution if the treatment is aimed at improving or preserving the state of health of those subject to it, but also at preserving the state of health of others, since it is precisely this further purpose, pertaining to health as an interest of the community, which justifies the compression of that self-determination of man which is inherent in the right of everyone to health as a fundamental right”* (16).

Similarly, in 1994, the same Court held that the protection of collective health “*implies and includes the duty of the individual not to damage or endanger the health of others through his or her own behavior, in observance of the general principle that each person's right is limited by mutual recognition and equal protection of the coexisting rights of others”* (17).

In that judgment, no. 218 of 2 June 1994, the Court declared unconstitutional Article 5 of Law no. 135 of 5 June 1990 on AIDS (18), which provided that no one could be tested for HIV infection without his or her consent except on the grounds of clinical necessity in his or her own interest.

In fact, the judges considered that the provision represented a prejudice to collective health, since “*Article 32 of the Constitution [...] implies [...] the duty to protect the right of third parties who come into necessary contact with the person for activities involving a serious risk, not voluntarily assumed, of contagion”* (17).

Another recent confirmation of the non-incompatibility of the imposition of compulsory vaccination with Article 32 of the Constitution came again from the Italian Constitutional Court in 2018.

With judgement no. 5 of January 18, 2018, the Italian Constitutional Court declared partly inadmissible and partly unfounded the question of constitutional illegitimacy raised by the Veneto region in relation to the vaccination requirement introduced by Law 119/2017 (transition from 4 to 10 mandatory vaccines for children from 0 to 16 years of age) (19).

The reasons for the judgement state: “*The law imposing a health treatment is not incompatible with Art. 32 Cost. This is the case if the treatment is intended not only to improve or preserve the state of health of the person undergoing it, but also to preserve the state of health of others; if it is provided that it does not adversely affect the state of health of the person who is obliged to undergo it, except only for those consequences that appear normal and, therefore, tolerable; and if, in the hypothesis of further damage, the payment of an equitable indemnity in favor of the damaged party is provided for, and this regardless of the parallel protection of compensation …”* (20).

Regarding the last sentence of the judgment extract, the reference is to Law 210/1992 (“Economic indemnity for persons affected by irreversible pathological impairment following compulsory vaccinations, transfusions, and administration of hemoderivatives”), which protects victims of permanent damage deriving from compulsory health treatments, offering them the possibility of receiving adequate financial compensation after an appropriate medical-legal evaluation (21).

Another constitutional principle of central importance in qualifying the imposition of compulsory health treatments is that set out in Article 2: “*The Republic recognizes and guarantees the inviolable rights of the person, both as an individual and in the social groups where human personality is expressed. The Republic expects that the fundamental duties of political, economic and social solidarity be fulfilled”* (15).

Article 2 enshrines the principle of social solidarity between the individual and the community, according to which the citizen, as a member of a community, is called upon to act not only for his own personal interests, but also to protect collective interests.

Thus, the combined provisions of Articles 32 and 2 of the Italian Constitution make the legitimacy of compulsory vaccination conditional on an appropriate balance between protecting the health of the individual and the community.

In the near future (since the official publication of the decree-law passed on 5 January 2022), the vaccine will be compulsory in Italy for all citizens over the age of 50.

Until 31 December 2021, the COVID-19 vaccine was compulsory in Italy for all healthcare professions and workers, pursuant to Article 4 of Decree-Law no. 44/2021 (22).

This was, in fact, a “selective” vaccination requirement, in that it was intended for a specific category of workers, and a “temporary” one, operating until 31 December 2021.

According to the provisions of the law, failure to comply with the vaccination requirement by those who “*carry out their activities in public or private health, social and health care and social assistance structures, in pharmacies or parapharmacies and professional offices”* results in the suspension of the right to perform services or tasks involving interpersonal contacts.

As a matter of fact, SARS-CoV-2 is classified as a human pathogenic agent of risk group 3) according to Art. 267 of Legislative Decree no. 81/2008 (the so-called “Unified Text on Occupational Safety and Health”), i.e., the category that “*includes pathogenic microorganisms that can cause disease in humans and pose a serious risk to workers; they can spread in the community but effective prophylactic or therapeutic measures are usually available”* (23).

In line with this principle, EU Directive 2020/739 of 3 June 2020 also included SARS-CoV2 among the biological agents against which protection in the workplace is mandatory (24).

On the basis of the combined provisions of Article 267 of Legislative Decree 81/2008 and Article 2087 of the Civil Code (which states that the employer is obliged to protect the physical integrity of employees), on 19 March 2021 the Court of Belluno issued an ordinance declaring legitimate the conduct of the management of a nursing home that had deemed unfit for duty and forced to take leave 10 healthcare workers who had refused to undergo the COVID-19 vaccine (25).

The ordinance, therefore, rejected the appeal filed by the claimants, who argued the constitutional illegitimacy of Article 4 of Decree-Law no. 44/2021 insofar as it obliged healthcare workers to vaccinate. The Court held that the question was manifestly unfounded, since the imposition of medical treatment aimed at protecting the health of others is entirely compatible with the Italian Constitution, provided that the subject is guaranteed fair compensation in the event of damage beyond normal foreseeability.

## Mandatory COVID-19 Vaccine: the Reasons for Controversy

The vast majority of civilized countries require their citizens to undergo a series of compulsory vaccinations starting from childhood.

In Italy, for example, all minors between zero and 16 years of age and unaccompanied foreign minors must undergo 10 vaccinations.

Children who are not up to date with vaccinations cannot access school services (19).

The imposition of compulsory health treatments such as childhood vaccinations was always accompanied by a lively bioethics debate, which however never reached even remotely the proportions of the dispute regarding the compulsory vaccine against COVID-19.

This is because COVID-19 vaccines have characteristics that make their mandatory imposition particularly controversial, chief among them the lack of final approval by regulators in many countries.

With particular reference to the European context, any pharmaceutical company wishing to market a drug within the European Union must first apply for marketing authorization by submitting an application to the European Medicines Agency (EMA).

Based on recommendations provided by the EMA, which carefully evaluates the drug efficacy and safety profiles, the European Commission can issue 3 types of authorization: emergency use authorization (EUA), conditional marketing authorization (CMA), and standard marketing authorization (SMA) (26, 27).

So far, the European Commission has granted four conditional marketing authorizations for vaccines developed by BioNTech and Pfizer, Moderna, AstraZeneca and Janssen Pharmaceutica NV, after the EMA gave a positive assessment of their safety and efficacy. The other vaccines are at different stages of evaluation.

Conditional marketing authorization is granted in cases where not all the clinical data for a drug required for standard authorization are available, but the benefit of placing the drug on the market immediately is considered to outweigh the risks related to the temporary incompleteness of the data.

Conditional marketing authorization is granted when 4 requirements are simultaneously met: there is a favorable benefit-risk ratio for the drug; all conditions are in place to believe that the pharmaceutical company will be able to provide complete data after authorization; the medicine meets an unmet medical need; and the benefit of the drug's immediate availability to patients outweighs the risk inherent in the fact that additional data are still needed.

Conditional marketing authorization is valid for 1 year and may be renewed.

The conditional marketing authorization imposes several obligations on the authorization holder that must be fulfilled within defined time frames, such as collecting additional data to demonstrate that the drug is effective and safe.

The marketing authorization can be converted to a standard marketing authorization once the marketing authorization holder meets the imposed obligations and complete data confirm that the drug's benefits continue to outweigh the risks.

The procedure for authorizing the marketing of a drug under the American regulatory authority, the FDA (Food and Drug Administration), has similar characteristics, but is carried out more quickly due to the implementation of streamlined procedures such as “fast track”, “priority review”, and “accelerated approval”.

This procedural simplification enabled the US FDA to grant final approval of the mRNA vaccine developed by BioNTech and Pfizer on 23 August 2021 for everyone over 16.

Until then, commercialization of the vaccine in the United States had been granted by virtue of an emergency use authorization dated 11 December 2020 (28).

The vaccines developed by Moderna and Janssen Pharmaceutica NV are still marketed in the US due to an emergency authorization issued by the FDA on 18 December 2020 and 27 February 2021, respectively.

The relatively unknown nature of the etiological agent responsible for the COVID-19 disease and the development of vaccines in an extraordinarily short timeframe make the described criticalities in the path to final approval of vaccines quite natural.

In any case, it should be noted that, regardless of the marketing approval process, the COVID-19 vaccine is the first drug in history to have benefited from a “real-life” test of exceptional proportions, having been administered to over 5.5 billion people and having shown absolutely satisfactory efficacy and safety profiles.

Regarding safety, according to EMA data, as of 28 October 2021, 412,571 adverse effects have been reported in 428,000,000 doses of Comirnaty vaccine administered to European citizens (0.09%), 214,528 in 68,800,000 doses of Vaxzevria (0.31%), 94,636 in 61,600,000 doses of Spikevax (0.15%) and 28,244 in 16,300,000 doses of Janssen (0.17%).

The vast majority of recorded adverse effects were mild or moderate (29).

Regarding efficacy, although COVID-19 vaccines show relatively modest effectiveness in preventing the contraction of viral infection (30, 31), their overall ability to control the onset of serious illness requiring hospitalization and intensive care has been proven by the world's most authoritative clinical studies (32–35).

Important decisions on compulsory vaccination against COVID-19 have been taken within the European institutions.

The Council of Europe, the main international organization committed to protecting human rights, separate and independent from the European Union, signed Resolution no. 2361 on 27 January 2021 (“Covid-19 vaccines: ethical, legal and practical considerations”).

The text clearly rules out the possibility of individual states making the COVID-19 vaccine compulsory and prohibits its use as a means of discrimination.

In points 7.3.1 and 7.3.2, the Resolution requires member states to: “*… ensure that citizens are informed that the vaccination is not mandatory and that no one is under political, social or other pressure to be vaccinated if they do not wish to do so; ensure that no one is discriminated against for not having been vaccinated, due to possible health risks or not wanting to be vaccinated”* (36).

However, this Resolution, being issued by the Parliamentary Assembly of the Council of Europe, is not a source of law, and is therefore neither binding nor mandatory for individual member states.

A possible conflict between the domestic law of one of the European States and the Council of Europe Resolution never implies illegality of the national rules.

This is not the case for the judgments of the European Court of Human Rights, which is called upon to check whether national laws comply with the principles laid down in the European Convention for the Protection of Human Rights and Fundamental Freedoms.

The Strasbourg Court has so far ruled on cases related to the COVID-19 pandemic on three occasions.

In the first case (Le Mailloux v. France, 5 November 2020, declaration of inadmissibility), concerning a French citizen who claimed that national legislation had failed to comply with the positive obligations to protect life and health of persons enshrined in Article 2 ECHR by not providing citizens with adequate means of defense against the spread of the virus (masks and tests), the Court dismissed the application because the applicant did not have “victim” status (37).

The second case concerns a Romanian citizen's appeal against the imposition of lockdown, which allegedly violated Article 5 of the ECHR, protecting personal freedom (Terheş v. Romania, 13 April 2021, declaration of inadmissibility).

The Court dismissed the appeal because the lockdown does not impose restrictions that can be regarded as a “deprivation of liberty” within the meaning of Article 5 ECHR (38).

The third case concerns an application for provisional measures made by 672 French firefighters, who invoking Articles 2 and 8 of the ECHR asked the Strasbourg Court to suspend as an interim measure the provisions of the French law no. 2021–1040 of 5 August 2021 imposing on their category the vaccination requirement to work (Abgrall and 671 Others v. France, 24 August 2021, rejection of requests for interim measures).

The Court rejected the appeal of the 672 firefighters as being outside the scope of Article 39 of the Rules of Court, which governs the conditions for adopting interim measures (39).

Indeed, the Court stated that granting interim measures is possible only in exceptional circumstances, when the applicants would otherwise face “a real risk of irreversible harm”.

However, it must be emphasized that this judgment excludes the existence of conditions suitable for the adoption of emergency protective measures, and in no way precludes the possibility that the Court may subsequently declare the admissibility of the firemen's action concerning the compatibility of the French legislation with the principles of the ECHR.

In summary, therefore, in none of the three decisions of inadmissibility the Strasbourg Court tackled head-on the question of the legitimacy of compulsory vaccination.

## How to Enforce a Potential Obligation?

Another central issue regarding the actual applicability of a direct vaccination obligation to all nation citizens concerns how this obligation would be enforced.

Basically, two compulsory vaccination policies are conceivable.

The first consists of a highly coercive strategy, a “hard” compulsory vaccination, whereby the drug is administered against the individual's will through the intervention of law enforcement.

The second, decidedly softer, option is to bar people who have not been vaccinated from participating in social and working life by adopting a vaccination passport.

The policy of forced inoculation presents countless critical elements in its hypothetical application and must therefore be considered as merely abstract.

On the other hand, the vaccine passport strategy is far more feasible and is in fact already partially applied in EU countries.

The application is “partial” because not only vaccinated citizens, but also citizens who have recovered or tested negative to a molecular swab carried out within the last 72 h can obtain the EU digital COVID certificate.

Shifting from a partial application of the digital COVID certificate to an extensive application, i.e., a vaccination passport granted only to those who have been vaccinated, would in fact represent the imposition of a vaccination obligation.

However, according to this provision, there would be a thorny new issue to be addressed, that of the actual usefulness of vaccinating people who have recovered from COVID-19, and are therefore naturally immunized.

Scientific evidence suggests that healed individuals with adequate antibody levels are more protected from reinfection than vaccinated people (40, 41).

Vaccination against COVID in recovered individuals may even be burdened with a higher probability of adverse effects (42, 43).

In accordance with these scientific data, it would not be unreasonable to grant vaccination passports not only to those who have been vaccinated, but also to those who can prove that they have recovered from the infection, as is the case in Switzerland (44).

However, it should be noted that the introduction of a vaccine passport as a prerequisite for access to social and working life would have a paradoxical effect, i.e., it would exacerbate restrictions on the personal freedom of the population instead of restoring the freedoms of all (the primary objective of vaccination).

In the light of this reflection, the choice of basing the compulsory vaccination policy on the immunity passport would therefore be counterproductive.

This would open up a third way in which the compulsory vaccination could be enforced: the imposition of fines on individuals who do not wish to be vaccinated.

The idea of creating a specific offense and the related criminal consequences (arrest and imprisonment) to punish those who do not intend to undergo vaccination is to be discarded, for the simple reason that no judicial system would be able to withstand the impact of such a measure. Italy, for example, had around 5.5 million unvaccinated people at the beginning of 2022, for which an equal number of criminal prosecutions should be initiated.

A financial penalty for those who do not comply with the vaccination requirement would be much more feasible.

This fine, however, to fulfill the task at hand, should be of such a magnitude as to have a substantial impact on the person's economic status.

In other words, a system of economic penalties that provides for monetary sanctions commensurate with the income of the person sanctioned would be effective, as is already the case in some countries (Switzerland and Finland).

In Italy, the size of economic sanctions is not related to the financial resources of the individual, and the definition of a fixed monetary amount as a fine to be paid in case of non-compliance with the vaccination obligation would lead to an obvious social inequity, with rich people willing to pay in order to preserve their non-vaccinated status and poor people forced to comply with the legal obligation.

## Conclusion

The alarming rate of progression of the fourth wave of the COVID-19 pandemic, particularly in Europe, has placed the issue of compulsory vaccination at the center of the international legal and bioethical debate.

As shown by the brief collection of principles enshrined in international treaties and jurisprudential pronouncements proposed, the right to individual self-determination is not configurable as intangible, being subordinate to the duty to ensure public safety.

In this sense, in accordance with the legal guidelines outlined above, we consider the legal bases for imposing a generalized vaccination obligation to be sufficiently sound.

Obviously, such an obligation must be based on reliable scientific data attesting to the absolute safety and efficacy of the COVID-19 vaccine.

Although authorisations for these vaccines are still largely conditional (only in the United States has one vaccine obtained final approval), it cannot be forgotten that more than 2 years after the pandemic broke out, SARS-CoV2 is putting even the most advanced health systems in serious difficulty.

Vaccines have proved to be an extraordinarily effective tool in containing the spread of the infection and limiting hospitalisations and deaths.

Their safety and efficacy have been widely proven in studies carried out all over the world.

These safety and efficacy profiles have enabled these drugs to obtain conditional approvals from the major regulatory agencies. These authorisations, although “conditional”, were granted after a thorough and scrupulous process of verifying the existence of a benefit-risk balance in favor of the benefits.

As for adverse events, although their existence is undeniably documented, it is absolutely impossible to imagine that a worldwide vaccination campaign could result in an absolute absence of undesirable effects.

Although it may therefore seem anomalous to impose a compulsory requirement for drugs that have not yet been definitively approved, in our opinion the extraordinary and exceptional nature of the pandemic situation makes it fully justifiable.

In our view, waiting for the final authorisations to be granted before imposing compulsory vaccination would pose a serious danger of delay in the fight against the fourth pandemic wave.

## Author Contributions

FG: conceptualization and writing original draft. GR, AS, and DD: writing, reviewing, and editing. All authors contributed to the article and approved the submitted version.

## Conflict of Interest

The authors declare that the research was conducted in the absence of any commercial or financial relationships that could be construed as a potential conflict of interest.

## Publisher's Note

All claims expressed in this article are solely those of the authors and do not necessarily represent those of their affiliated organizations, or those of the publisher, the editors and the reviewers. Any product that may be evaluated in this article, or claim that may be made by its manufacturer, is not guaranteed or endorsed by the publisher.
